# Component Analysis and Free Radicals Scavenging Activity of *Cicer arietinum* L. Husk Pectin

**DOI:** 10.3390/molecules15106948

**Published:** 2010-10-11

**Authors:** Vania Urias-Orona, Joselina Huerta-Oros, Elizabeth Carvajal-Millán, Jaime Lizardi-Mendoza, Agustin Rascón-Chu, Alfonso A. Gardea

**Affiliations:** 1 Centro de Investigación en Alimentación y Desarrollo, A.C., Carretera a la Victoria Km 0.6, Hermosillo, Sonora, Mexico; 2 Centro de Investigación en Alimentación y Desarrollo, A.C., Faculty of Human Nutrition, Autonomous University of Nuevo León, Nuevo León, Mexico; 3 Centro de Investigación en Alimentación y Desarrollo, A.C., Carretera al Varadero Nacional Km 6.6, Col. Las Playitas 85480 Guaymas, Sonora, Mexico

**Keywords:** : *Cicer arietinum*, pectin polysaccharide, FTIR, antioxidant activity

## Abstract

A pectin (CAP) was extracted from the husk of *Cicer arietinum* L.. Monosaccharide analysis of CAP revealed the dominance of galacturonic acid and smaller amounts of galactose, arabinose, rhamnose, glucose, xylose and mannose. Viscosimetric analysis showed that the intrinsic viscosity ([*η*]) and the molecular weight (MW) of CAP were 296 mL/g and 105 kDa, respectively. The degree of esterification (DE = 10%) was determined by FTIR spectroscopy. CAP exhibited a dose-dependent free radical scavenging activity, as shown by its DPPH radical inhibition. At 1.0 mg/mL CAP exhibited a scavenging rate of 29% on DPPH radicals. The evaluation of antioxidant activity suggested that CAP had good potential for DPPH radical scavenging activity and should be explored as a novel potential antioxidant.

## Abbreviations

CAP: *Cicer arietinum* L. pectin
MW: molecular weight
DPPH: 2,2-diphenyl-1-picrylhydrazyl
[*η*]: intrinsic viscosity
HM: high methoxy
LM: low methoxy
FTIR: Fourier transform infrared spectroscopy
DE: degree of esterification

## 1. Introduction

Many human diseases are accompanied by activation of free radicals, considered a mechanism of biological membrane destruction. Free radicals induction occurs during inflammatory processes, cardiovascular problems, cancer, allergic reactions, among other diseases. The endogenous antioxidant system protects cells and tissues against free radicals. However, these endogenous antioxidants cannot totally prevent the development of oxidative stress. Therefore, it is of interest to search for some exogenous substances with antioxidant properties that might be used as prophylactics or therapy of free radicals activated diseases [1].

Polysaccharides and their conjugates, used for a long time in the food and medicine industries, have attracted much attention due to their biological activities. It has been reported that polysaccharides in general have strong antioxidant activities and can be explored as novel potential antioxidants [2]. Due to their antioxidant activity, polysaccharides extracted from fungal, bacterial and plant sources have been proposed as therapeutic agents [3]. It has been recently reported that pectins and pectic acids present antioxidant activity, which has been related to the reduction power and free radical scavenging activity of these molecules [2,4,5]. 

Pectin is a structural polysaccharide component of cell walls. This is composed of a backbone of (1→4)-linked α-D-galacturonic acid units. The homogalacturonic regions are interrupted by rhamnogalacturonic regions containing (1→2)-linked α-L-rhamnopyranosil residues. Rhamnosyl units can be substituted by side chains containing arabinose and galactose. Galacturonic acid residues can be partially esterified by methanol on the carboxyl group [6]. According to the degree of methoxylation (DM), pectin is divided into high methoxy (HM) pectin with DM > 50% and low methoxy (LM) pectin with DM < 50%. The DM determines the functional properties of pectins, which are the base of their broad use [7]. 

Although most plant tissues contain pectin, citrus and apple peel are the major sources of pectic substances around the world [7]. On the other hand, a large amount of chickpea (*Cicer arietinum* L.) by-products is produced during processing in regions where this is a major food legume (Southern Europe, North Africa, India and Middle East countries). The majority of chickpea processing wastes include chickpea husk [8], which is used for animal nutrition. A previous research indicated that chickpea husk could be a source of pectic substances [9]. However, to our knowledge, detailed information on the physicochemical properties and antioxidant activity in pectin from *Cicer arietinum* L. husk has not been yet reported elsewhere.

The aim of this study was to investigate the physical-chemical properties and *in vitro* antioxidant capacity of *Cicer arietinum* L. husk (CAP) pectin in order to define the potential usefulness of this biomaterial as a functional food.

## 2. Results and Discussion

### 2.1. Physical-chemical properties

The pectin extracted from chickpea (CAP) represents 9% of the dry tissue weight. CAP pectin obtained consists of 67% (w/w) of galacturonic acid. Galactose, arabinose and rhamnose were the main neutral sugars components in CAP. Glucose, xylose and mannose were minor and fucose was not present (Table 1). This profile could be related to the presence of diverse species of arabinogalactans in the branched regions that are inserted along the homogalacturonan chain. These results are consistent with those reported before in pectin from *Cicer arietinum* epicotyl cells [10,11]. 

**Table 1:** Sugar composition (%) of *Cicer arietinum* L. pectin.

Galacturonic acid	69.0 ± 0.5
Galactose	10.5 ± 0.7
Arabinose	6.9 ± 0.5
Rhamnose	6.1 ± 0.6
Glucose	1.2 ± 0.3
Xylose	0.9 ± 0.1
Mannose	0.4 ± 0.1

All results were obtained from triplicate experiments.

CAP presented an intrinsic viscosity ([*η*]) of 296 mL/g. The viscosimetric molecular weight (M*v*) of CAP was 105 kDa, which is in the range reported for pectin extracted from citrus peel (50 to 2,000 kDa) [12] and higher than those indicated in LM pectin like citrus and olive pomace pectin (51 and 14 kDa) [13]. 

**Figure 1:**
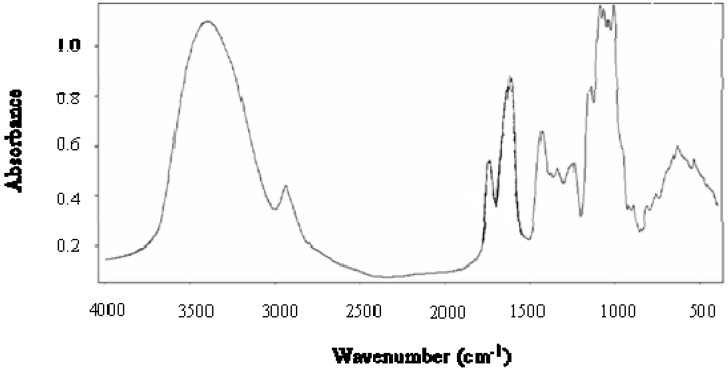
FTIR spectrum of *Cicer arietinum* L. pectin.

The Fourier transform infrared (FTIR) spectrum of CAP is presented in Figure 1. According to several authors [14,15], three regions can be distinguished: a region from 3,500 to 1,800 cm^-1^, with two peaks (3,350 cm^-1^ corresponding to stretching of the OH groups and 2,950 cm^-1^ corresponding to the C-H stretching of the CH_2_ groups). A region from 1,800 to 1,500 cm^-1^, with three peaks associated with the carboxylic acid (COO-), the methyl ester (COOCH_3_) and the primary amide groups (CO-NH_2_). The last region below 1,500 cm^-1^ is considered as the ‘fingerprint’ for a given compound and corresponds principally to coupled C-C, C-O-C and C-OH vibration modes of the carbohydrate ring and to the glycosidic linkage vibrations. The FTIR spectrum of CAP shows a lower absorbance at 1,750 cm^-1^ than at 1,650 cm^-1^, indicating a LM pectin. These data were used to calculate the degree of esterification (DE) of CAP [14] obtaining a value of about 10%.

### 2.2. Antioxidant activity

**Figure 2:**
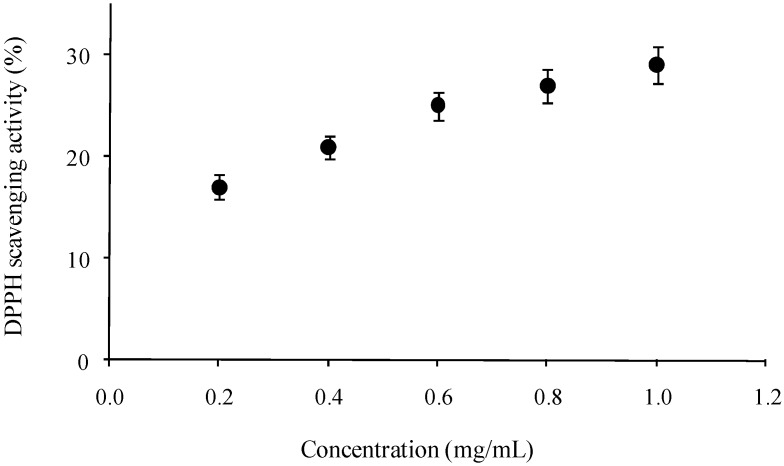
Scavenging activity of *Cicer arietinum* L. pectin against DPPH radicals. Each value is presented as mean ± standard error (n = 3).

The antiradical performance of CAP with respect to DPPH radicals was measured. DPPH radicals have been widely used as a model system to study the scavenging activity of different natural compounds. The color of the system change from purple to yellow when the absorbance at 517 nm decreases as a result of the formation of DPPH-H through donation of hydrogen by antioxidants [16]. Figure 2 shows that the scavenging activity of CAP on DPPH radicals is related to the polysaccharide concentration. At the concentration of 1.0 mg/mL, the antioxidant activity was 29%, which is higher than those of commercial pectin (around 10%) with different DE (25, 65 and 94%), under the same concentration [5]. However, the scavenging activity of CAP against DPPH was much lower than that reported for pectic acid (60%) under the same concentration (1.0 mg/mL) [4]. It has been suggested that pectin interacts directly with oxidants and free radicals [17]. The antioxidant activity in CAP could be related to the high galacturonic acid content. It has been reported that a relatively low molecular weight and a high uronic acid content in polysaccharides appeared to increase the antioxidant activity. However, the mechanism of free-radical scavenging of polysaccharides is still not fully understood [18]. 

## 3. Experimental

### 3.1. Materials

A chickpea sample (Suprema variety) was kindly provided by the National Institute for Investigation in Forestry, Agriculture and Animal Production in Mexico (INIFAP-CEVACU). All chemical products were purchased from Sigma Chemical Co. (St Louis, MO, USA).

### 3.2. Methods

#### 3.2.1. Pectin extraction

Chickpea seeds were first heated (1 kg seeds/2 L water) for 15 min at 50 ºC. Husks were then manually separated, dried at 40 ºC overnight and milled to 0.84 mm particle size. Milled husks were dispersed in 0.05 M citrate phosphate buffer at pH 5 (100 g/600 mL) and treated enzymatically for starch and protein degradation, using α-amylase solution (Termamyl®120, pH 7, 100 ºC, 30 min, 75 U/g sample), amyloglucosidase, (2 h, 60 ºC, pH 5.5, 240 U/g sample, 80 rpm) and pronase (pH 7 at 25 ºC to 18 h, followed for 100 ºC, 10 min, 0.4 U/g sample). Pectin extraction was performed twice under acid conditions, using 0.05 N HCl (1:6) at 80 ºC for 1 h and 80 rpm and both supernatants were collected. The extract was centrifuged at 12,040 × g for 10 min and the pH adjusted to 3.5. The extract was dispersed into 3 volumes of 96% ethanol during 1 h at 4 ºC in order to precipitate pectin, which was then collected by filtration through 4 µm (Whatman Chemical Separation, Inc., Clifton, NJ, USA) and freeze-dried.

#### 3.2.2. Sugar composition

Sugar composition was determined after pectin hydrolysis with 2 N trifluoroacetic acid at 120 ºC for 2 h. The reaction was stopped on ice, the extract was evaporated under air at 40 ºC and rinsed twice with water (200 μL) and resuspended in water (500 μL). All samples were filtered through 0.45 μm (Whatman) and analysed by high performance liquid chromatography (HPLC) using a Supelcogel Pb column (300 × 7.8 mm; Supelco, Inc., Bellefont, PA) eluted with 5mM H_2_SO_4_ (filtered 0.2 μm, Whatman) at 0.6 mL/min and 50 ºC [19]. A Varian 9012 HPLC with Varian 9040 refractive index detector (Varian, St. Helens, Australia) and a Star Chromatography Workstation system control version 5.50 were used. A series of sugar calibration standards was prepared in HPLC grade water at concentrations appropriate for creating a calibration curve for each sugar of interest (galacturonic acid, arabinose, galactose, rhamnose, xylose, mannose and glucose) in the range of 0.2–12.0 mg/mL. The internal standard was inositol. 

#### 3.2.3. Degree of esterification (DE)

The analysis was performed by FTIR spectroscopy (Nicolet Instrument Corp. Madison, WI, USA). All standards and samples were dried and stored in desiccators prior to FTIR analysis. Samples were incorporated into KBr and pressed into a 1 mm pellet. A calibration curve was developed for DE determination by using pectin commercial standards with a known degree of esterification. DE value of CAP was calculated using linear fit equation of calibration curve obtained by correlation of the ratio: area of esterified carboxyl groups/(area of esterified carboxyl groups + area of nonesterified carboxyl groups) of pectin commercial standard with their corresponding known DE value [14].

#### 3.2.4. Intrinsic viscosity

Specific viscosity, *η*_sp_ was measured by registering pectin solutions flow time in an Ubbelohde capillary viscometer (OB size; Koehler Instrument; USA) at 25 ± 0.1 ºC, immersed in a temperature controlled bath. Pectin solutions were prepared in the concentration range from 0.1 to 20 g/L, dissolving dried pectin in an aqueous solution containing 0.1 N NaCl at pH 7 for 18 h with stirring at room temperature. Pectin solutions and solvent were filtered using 0.45 µm membrane filters before viscosity measurements. The intrinsic viscosity ([*η*]) was estimated from relative viscosity measurements, *η*_rel_, of pectin solutions by extrapolation of Kraemer and Mead and Fouss curves to “zero” concentration. NaCl was used in order to prevent pectin aggregation.

#### 3.2.5. Viscosimetric molecular weight

The viscosimetric molecular weight (*Mν*) was calculated from the Mark–Houwink relationship, *Mν* = ([*η*] */ k*)^1^*^/α^*, where the constants *k* and *α* are 0.0436 and 0.78, respectively.

#### 3.2.6. DPPH scavenging activity

The antioxidant activity was assessed as described before [20]. A 0.1 mM solution of DPPH (2,2-diphenyl-1-picrylhydrazyl) in ethanol was prepared and this solution (1 mL) was added to sample (10–50 μg/mL, 2 mL) in water. After 30 min, absorbance was measured at 517 nm. Vitamin C was used as a positive control. The inhibition of DPPH radicals by the pectin samples was calculated according to the following equation:

DPPH-scavenging activity (%) = [1-(A_sample517nm_-A_blank517nm_)/ A_control517nm_] × 100

The measurements were performed using a spectrophotometer Lambda 25 UV/VIS (PerkinElmer, USA).

#### 3.2.7. Statistical analysis

All determinations were made in triplicates and the coefficients of variation were lower than 8%. All results are expressed as mean values.

## 4. Conclusions

The results of the present study indicate that the low methoxy pectin obtained from the husk of *Cicer arietinum* L. exhibit antioxidant activity, which could be related to the galacturonic acid content. The present finding provides a basis for further structural analysis and evaluation of the bioactivities of the chickpea pectin for its application in food and medicinal fields.
